# Tightening Loose Plates

**Published:** 1909-09-15

**Authors:** F. G. Worthley


### TIGHTENING LOOSE PLATES.

BY F. G. WORTHLEY, D.D.S.

Since the introduction of rubber dentures (and long before for that matter, dentists have wished for a practical method of tightening plates that have become loose through resorption of the underlying tissues. Also the correcting of defects in slightly misfit plates without going through the entire operation of remaking the plate. Especially in cases where a plate has been worn for some time and the wearer has become accustomed to it and to the expression it gives to the face, it is difficult, and generally impossible, to make a new denture which will prove as satisfactory to the wearer as the old. Moreover a method which will eliminate the tedious technic of taking an impression, making a model and securing a proper bite, and will give as good or better results, is certainly a desideratum. The method described below is applicable to all of the conditions named, but perhaps finds its greatest field of usefulness in the resetting of so-called temporary plates.

Probably there are few dentists of to-day who fail to appreciate the esthetic value of temporary plates. The atrophy of the expressional muscles, the undue resorption of the alveolar process, the pronounced change in expression which follows the extraction of all the teeth are in a large measure avoided by the immediate insertion of a denture.

Formerly the bugbear of a temporary plate was the fact that the resorption of the tissues under it soon permitted it to become so loose that it acted as an irritant by chafing the tissues or could not be retained in position at all. To overcome this attempts were made to reline and refit the plate by putting in a layer of new rubber to compensate for the resorption. Soft modeling compound or plaster was placed in the plate and it was pressed to place and an impression taken in that manner. The case was then flasked and after separation the layer of impression material was removed and rubber substituted. In some cases, in which the ridge was slight with no undercut above, this method was fairly successful. In the great majority of cases the method was not practical for the reason that where the ridge was at all prominent the model could not be drawn from the rigid plate without fracture.

The present method is to take the impression directly in the rubber which, when vulcanized, will become an integral part of the plate. For this purpose several forms of plastic, paste and liquid rubbers have been devised.

In the hands of the writer the paste preparation, sold under the name of "Bridgford's plate paste," has proved most satisfactory. It is put up in collapsible tin tubes, is cleanly to handle and of a consistency which takes a sharp impression.

In taking an impression with any plastic material in an old plate the chief point to be observed is to so distribute the material as to make a thin film without using undue pressure. When using the paste mentioned above the following, technic will give satisfactory results:

Having thoroughly cleaned the plate, particularly on the palatal side, it is dried and a quantity of the paste squeezed from the tube upon the palatal surface. This is sprea dover the surface with a wet spatula. The proper quantity to use is a matter of judgment and experience. The beginner is apt to use too little rather than too much. The plate is placed in the mouth and pressed to place with slow and gentle pressure. If too much pressure is used the material tends to stick to the mucous surface. It is better to use too little than too much pressure. The plate is removed and the surplus material that has been squeezed out around the edges is trimmed off. The plate is dipped in water and replaced in the mouth, using the same slow, careful pressure. This is repeated until but a thin layer of the paste covers the plate and shows a sharp impression of the mouth. At the last fitting the patient is instructed to close the jaws and go through the motions of chewing. This will correct any slight defects in articulation. The impression is allowed to dry and should then receive a coating of liquid silex. If a relief is desired it can be cut at this time with a sharp knife. The case may then be vulcanized. To do this it is only necessary to make a slight flasking. Fill a flask with plaster, bury the plate in it, and when hard, vulcanize. A little finishing around the edges is all that is necessary to complete the vulcanized plate.

As about all the time actually consumed in the operation is in taking the impression (generally about ten minutes)

it will be seen that a temporary plate may be refitted several times during the process of resorption without undue expense to the patient or loss of time to the dentist. When making a second refitting the old layer can be cut out to avoid making the plate too thick.

There are many cases of plates which do not fit accurately owing to a slight springing, warpage, defect in articulation or other cause which can be corrected in this manner with just as good results, and much less work than by making a new plate.

If the plate does not extend back far enough, or is not high enough on the rim at any point, it can be extended by pasting on warm vulcanizable gutta-percha, chilling it and permitting the paste to line it the same as the rest of the plate.—*Western Journal.*

